# Is the degree of facial swelling after dental extraction sufficient to justify the current delays to radiotherapy mask production? A pilot evaluation of postextraction swelling using 3D photography

**DOI:** 10.1002/cre2.540

**Published:** 2022-02-14

**Authors:** Robert McCormick, John Meechan, James Adams, Helen Stanncliffe, Lee Merecer, Kate Best, Michael Nugent

**Affiliations:** ^1^ Department of Oral and Maxillofacial Surgery Newcastle Upon Tyne Hospitals NHS Foundation Trust Newcastle Upon Tyne UK; ^2^ Newcastle University Faculty of Humanities and Social Sciences Newcastle Upon Tyne UK; ^3^ Present address: Lee Merecer, Department of Oral and Maxillofacial Surgery South Tees Hospitals NHS Foundation Trust Middlesbrough UK; ^4^ Present address: Michael Nugent, Department of Oral and Maxillofacial Sugery Sunderland Royal Hospital Sunderland UK

**Keywords:** 3D photography, dentoalveolar surgery, face‐mask, head and neck cancer, radiotherapy

## Abstract

**Background:**

Concern that facial swelling after dental extractions will spoil the fit of radiotherapy masks in head and neck cancer patients leads to the current practice of delay making of mask production (and therefore the start of radiotherapy) for several days or longer. However, there is little data on how extensive facial swelling is after dental extraction.

**Aim:**

To assess the degree of facial swelling in a group of adult patients attending Newcastle Dental School for routine dental extractions.

**Materials and Methods:**

Seventeen dental extraction patients underwent three‐dimensional photography using the 3dMDFace® system at 1‐week preop, immediately preop, and at 48‐h postop. We recorded demographic data, teeth extracted, and methods. Facial volume change was assessed using 3dMD Vultus® software. Two reviewers ran the data through the 3dMD Vultus® software independently. We used Student's *t*‐test to assess significance.

**Results:**

Twelve patients were included in the final analysis. There was no significant difference in the difference between the two preoperative measurements and the preoperative versus postoperative difference (Wilcoxon signed‐rank test: Reviewer 1: *p* = .31. and Reviewer 2: *p* = .10). Thus, mean facial swelling was less than the threshold for significant swelling which was deemed to be 15 cm^3^.

**Conclusion:**

Facial swelling following dental extraction may not be sufficient in itself to justify the current delays in mask production and subsequent delivery of radiotherapy. Further definitive studies are needed to optimize how dental extractions should be timed within head and neck cancer care pathways.

## 1 INTRODUCTION

### 1.1 Research problem

The study objective was to find out the degree of facial swelling following dental extractions (exodontia). The observation that swelling is minimal is significant for patients undergoing exodontia before radiotherapy for head and neck cancer as this may have an impact on how promptly radiotherapy can be delivered.

### 1.2 Background

The current national standard within the United Kingdom recommends that curative head and neck cancer irradiation should commence within 4 weeks of a decision to treat or within 42 days following surgery (British Association of Head and Neck Oncologists, 2009; Schache et al., 2021). Many patients who undergo radiotherapy require exodontia before treatment. Long‐term effects of radiotherapy include xerostomia, osteoradionecrosis, and trismus. Xerostomia increases the risk of periodontal disease and caries. Trismus reduces accessibility for dental care and treatment. Dental disease and dental extractions increase the risk of osteoradionecrosis, one of the most devastating long‐term effects of radiotherapy. For these reasons, extraction of teeth with a poor prognosis is recommended before radiotherapy.

However, there is evidence to suggest that radiotherapy is often delayed, particularly when exodontia is required. Only 58% of patients requiring exodontia before radiotherapy commenced treatment within 4 weeks of a decision to treat in a local retrospective audit compared with 91% of patients who did not require exodontia (Steele & Nugent, 2011).

**Figure 1:**
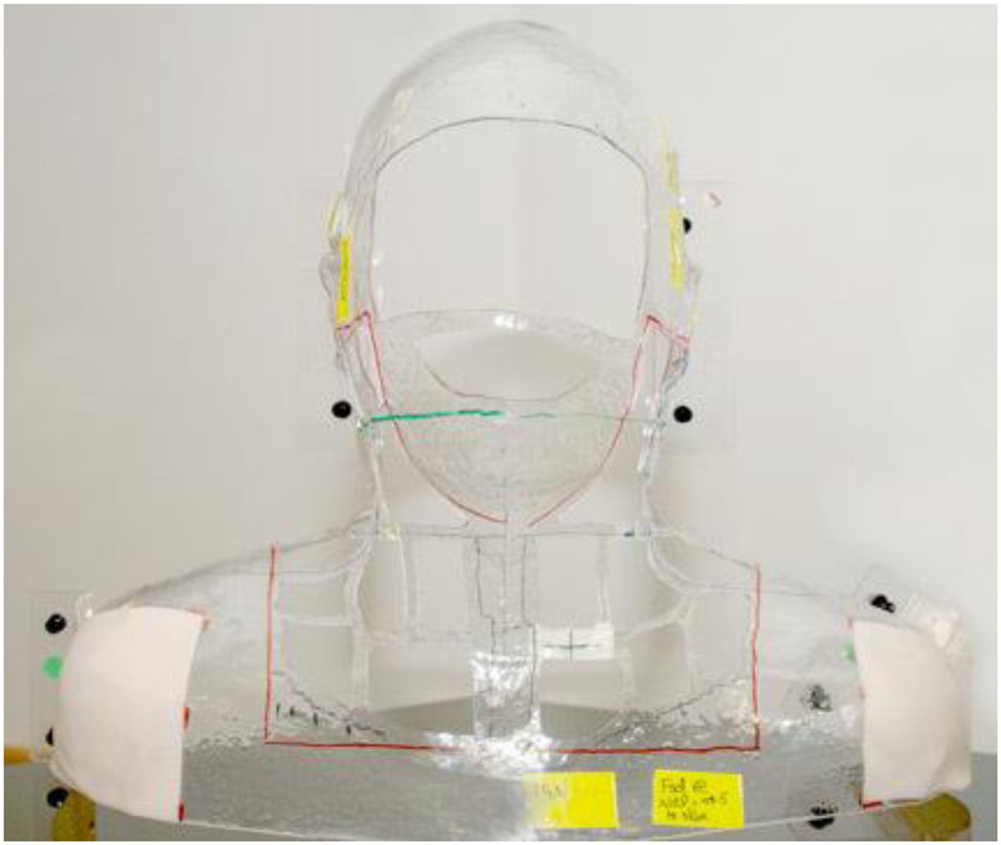
A face mask typically worn by head and neck cancer patients undergoing radiotherapy

The safe and accurate delivery of fractionated radiotherapy to the head and neck requires the manufacture of a customized face mask from impressions, allowing patients to be accurately positioned for treatment sessions (see Figure 1). The current practice in the United Kingdom is to delay face mask production following exodontia to prevent inaccurate fit. This is based on the current perception that dental extraction causes significant swelling, requiring several days to settle. However, delaying mask production potentially delays cancer treatment.

A number of methods to assess facial dimensional change have been investigated in the literature including ultrasound (Holland, 1979), facebow (Holland, 1979), frontal photography (Amin & Laskin, 1983), computed tomography scanning (Esen et al., 1999), and the facial plethysmograph (Milles et al., 1985). However, not all of these methods measure facial swelling volumetrically. Three‐dimensional (3D) photography (stereophotogrammetry) and laser scanning are more contemporary methods (Kau et al., 2006; Mocan et al., 1996). Modern 3D photographic systems are noninvasive, rapid, accurate, and reproducible methods of obtaining measurements of facial volumetric changes (Aldridge et al., 2005). The 3dMDface® System (3dMD, LLC., 2021) works by projecting random light patterns on the subject (face). It captures images in 2 ms with multiple synchronized digital cameras set at various angles. Algorithms developed by 3dMD integrate the various images obtained to produce a single 3D image. The resultant 3dMD image in conjunction with the measurement software has been verified to consistently record a geometric accuracy of <0.2 mm root mean square (Nord et al., 2015; Ullah et al., 2015).

Most studies that have looked at postextraction swelling have used techniques that measure soft tissue changes in just one or two dimensions, had limited accuracy, or where measurements were poorly reproducible (Markiewicz et al., 2008). There is some work in the literature regarding the assessment of soft tissue swelling with more reliable methods of measurement. These studies were on patients undergoing surgical removal of wisdom teeth or orthognathic surgery. The aim of these studies was often to examine the effects of drugs and other treatments at reducing postoperative swelling (Agostinho et al., 2014; ElHag et al., 1985; Holland, 1979; Ibikunle et al., 2016; Markiewicz et al., 2008; Milles & Desjardins, 1993; Mocan et al., 1996; Pappalardo et al., 2007; Saravanan et al., 2016). Few studies use 3D imaging for measuring facial swelling (Asutay et al., 2018; Matsuda et al., 2016). Thus, there is little or no research measuring facial swelling following routine dental extraction.

The purpose of this study is to evaluate the changes in the facial soft tissue volume, following routine exodontia, using 3D facial photography.

## 2 METHOD

We undertook a prospective pilot study. The primary outcome measure was the facial volumetric change in patients who underwent exodontia at Newcastle Dental Hospital using stereophotogrammetry (the 3dMDface system). Ethical approval was granted by the National Research Ethics Service, Newcastle and North Tyneside 1 (reference 11/H0906/12).

Inclusion criteria: Adult patients (18 years and over) attending Newcastle Dental Hospital requiring routine exodontia were invited to participate in the study. Exclusion criteria: Patients with a bleeding disorder, angioedema, patients prescribed regular systemic corticosteroids, and those patients who were immunocompromised. Informed consent was obtained from all eligible patients participating.

**Figure 2:**
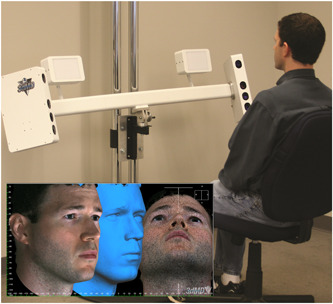
The patient positioning and set up of the 3dMD system (image courtesy of 3dMD)

Each participant underwent three sets of 3D facial imaging, using a standardized position (see Figure 2). One image was taken at least 2 days before exodontia (Photograph 1), the second immediately before but on the day of exodontia (Photograph 2), and the last taken 2 days postoperatively (Photograph 3). Taking three sets of photographs allowed patients to act as their own control. Taking the two preoperative photos at least 48 h would allow for potential daily fluctuations in facial volume. Each patient was placed in a standardized position for each image. Exodontia was performed by three staff surgeons in the oral surgery department.

3dMD Vultus®software (3dMD LLC, 2021; Atlanta, GA, USA) has been validated for use in the assessment of facial volumetric change following surgery (Ullah, 2014). This was used to assess volumetric changes between images. Two reviewers independently ran the volume change analysis protocol in Vultus as a means of data quality control. Other information collected included patients' characteristics, which teeth were extracted, and the volume and type of local anesthesia used. If exodontia required raising a mucoperiosteal flap, bone removal, or suture placement, this was recorded.

### 2.1 Sample size calculation

Calculation of the sample size required identification of the change of facial volume required to significantly affect the fit of a patient's customized face mask. No national standard tolerance is known. Therefore, we sought the opinion of our local medical physicists. A linear shift of 3 mm was decided to be significant. Assuming the mandible to be approximately triangular in axial section, and using standard mean measurements of facial size, an increase in one dimension of 3 mm, would represent a 15 cm^3^ change in facial volume. As mentioned above, since no previous study which looked at routine dental extractions was found, a study measuring facial swelling following surgical removal of wisdom teeth was used to calculate the standard deviation (Ullah et al., 2015). As surgical removal of wisdom teeth is more invasive compared to routine exodontia, the extent of swelling was expected to be greater. In this study, the control group's mean peak swelling was 26.0 cm^3^ (standard deviation 9.9 cm^3^). The group given steroids had a mean peak swelling of 17.7 cm^3^ (standard deviation 11.3), closer to the volume deemed significant for mask construction. It was reasonable to use this standard deviation to calculate the power of our study. Nine subjects gave an 80% chance of detecting a difference at the 0.05 level. Facial volume changes of 15 cm^3^ were considered significant.

### 2.2 Statistical analysis of results

In order to determine the significance of any measured change in facial volume, the difference in volume between Photograph 1 and Photograph 2 (*difference 1*), the difference in volume between Photograph 2 and Photograph 3 (*difference 2*) were calculated. A paired *t*‐test will then be performed to determine whether *difference 1* is equal to *difference 2*.

## 3 RESULTS

Seventeen patients were recruited to the study. Five patients were lost to follow‐up. Four of the five patients lost to follow‐up only attended Visit 1 (only having a baseline image taken) while one of the five attended for exodontia but failed to return for a postoperative image (Visit 3). Twelve patients were included in the study (complete follow‐up in 70.6% of patients). There was an even distribution of patients between the sexes included and the median age of recruits was 33 (interquartile range 23–52). Patient characteristics, procedure descriptions, and facial volume differences are detailed in Tables 1 and 2.

**Table 1:** Patient characteristics, procedure details, and facial volume measurements

Patient number	Age (years)	Gender	Teeth removed	Flap raised?	Bone removed?	Sutures	Volume difference (cm^3^) between			
							Photo 1 and 2		Photo 2 and 3	
							Reviewer 1	Reviewer 2	Reviewer 1	Reviewer 2
1		M	38	Y	Y	Y	2.898	−0.687	9.482	7.741
2	22	F	28	N	N	N	−0.252	−1.649	0.75	10.471
3	23	F	18	N	N	N	6.46	5.834	−2.75	1.8
4	56	M	17, 26, 27, 37, 46, 47	N	N	N	−3.711	−8.403	−3.675	1.829
5	71	M	Failed to return							
6	29	M	Failed to return							
7	71	F	Failed to return							
8	64	F	26, 35, 36	N	N	N	13	3.496	3.46	−10.563
9	19	M	18, 28, 38, 48	Y (for 38,48)	Y	Y	16.95	−6.079	22.8	33.041
10	29	M	28, 38	N	N	N	1.768	0.636	−0.269	−1.609
11	22	M	Failed to return							
12	34	M	38	N	N	N	1.267	9.348	2.1	1.038
13	33	F	48	Y	Y	Y	2.7	4.405	9.62	11.683
14	17	F	Failed to return							
15	25	F	48	Y	Y	Y	2.396	1.809	11.94	14.686
16	42	M	38	Y	Y	Y	2.52	−3.727	4.925	27.054
17	52	F	18	N	N	N	1.086	−0.993	2.96	6.27

**Table 2:** Facial volume changes as assessed per reviewer

Reviewer	Median difference between two preop volumes (cm^3^)	Median difference between preop and postop volume (cm^3^)
1	2.5 cm^3^ (95% CI: 1.2–4.7)	3.21 cm^3^ (IQR: 0.2–9.6)
2	−0.03 cm^3^ (95% CI: −2.7 to 4.0)	7.0 cm^3^ (95% CI: 1.4–13.2)

Abbreviations: CI, confidence interval; IQR, interquartile range.

There was “good” agreement in measurements between reviewers (intra‐class correlation: 0.63, 95% confidence interval: 0.12–0.88).

There was no significant difference in the difference between the two preoperative measurements and the preoperative versus postoperative difference (Wilcoxon signed‐rank test: Reviewer 1: *p* = .31 and Reviewer 2: *p* = .10).

## 4 DISCUSSION AND CONCLUSION

We have found that the increase in facial volume following exodontia was below the threshold for significance in the context of face mask production. Although there remains little published data on postdental extraction facial swelling, what data it is consistent with our findings. Matsuda et al. measured facial swelling following surgical removal of third molar teeth. They were testing the benefit of steroids on postextraction swelling. The mean increase in facial volume was 6.36 cm^3^ in the control group and 10.34 cm^3^ in the steroid group. They used a method similar to ours, albeit manufactured by a different company (Matsuda et al., 2016).

Another group used the 3dMD system to assess facial volume change following third molar removal. In their study, Asutay et al. were testing the efficacy of photobiomodulation (PBM) on postoperative swelling. Swelling peaked at Day 2. The mean swelling was 15.47 cm^3^ (±5.41) in the PBM group. They did not find a statistically significant difference in the groups. While the reported swelling is at the threshold of significance for our study, this was for surgical extractions, not routine ones (Asutay et al., 2018).

This would suggest that even in third molar surgery where swelling is significantly worse than for routine dental extraction it might not reach the threshold for interfering with mask fit.

Therefore, any swelling following dental extractions should not impact the accuracy of fit of patients' customized face masks, allowing precise delivery of radiotherapy regimens.

This study has some limitations. Our sample size was small. The study population is potentially not representative of head and neck cancer patients, who tend to be older and less fit. We had to estimate the threshold for swelling which would potentially interfere with mask fit. Definitive studies would need to empirically measure what degree of facial swelling interfered with mask fit. Our study attempted to determine if routine exodontia results in swelling which is significant enough to impact the safe delivery of radiotherapy. Although the majority of patients did undergo routine exodontia, five patients required more invasive techniques to facilitate exodontia such as the raising of a mucoperiosteal flap, bone removal, or suturing. Although not routine, these more invasive procedures are likely to result in swelling greater than expected following exodontia. The finding of nonsignificant swelling in a sample of patients, some of whom underwent more invasive techniques, is interesting. Two patients in our sample had beards (Patient 9 and 16). Anecdotally, it was felt that this made analysis of imaging more difficult when using 3dMD Vultus software and may have resulted in the higher than expected volume changes in these individuals. We would propose the presence of significant facial hair in the exclusion criteria or that patients shave before imaging when using 3D photography.

## 5 CONCLUSION

The findings of this pilot suggest that facial swelling after dental extractions is of the order of 10 cm^3^ or less. This raises the possibility that delaying mask production after dental extractions may be unnecessary and definitive studies investigating this would be warranted, addressing the weaknesses identified in this study.

## 6 CLINICAL RELEVANCE

### 6.1 Scientific rationale for the study

Head and neck cancer patients need to be dentally fit before starting radiotherapy. They also need to have a face mask made. Standard practice is to delay taking facial molds for several days after dental extractions, allowing any facial swelling to settle. This practice is not evidence based. New 3D photography techniques allow facial swelling to be measured.

### 6.2 Principal findings

This pilot study shows that postextraction dental swelling might not be sufficient to affect mask fit.

### 6.3 Practical implications

Radiotherapy mask production could happen without delay, although definitive studies would be needed.

## CONFLICT OF INTEREST

The authors declare no conflict of interest.

## AUTHORS CONTRIBUTIONS

**Robert McCormick**: Recruitment of patients, taking of photos, volume change analysis, preparation of the overall final document. **John Meechan**: Design of study, oversight of study, mentoring, overall preparation of the final document. **James Adams**: Initial concept, design, and writing of the background. **Helen Stanncliffe**: Recruitment of patients, development, and writing of methods section. **Lee Merecer**: Recruitment of patients, development, and writing of methods section. **Kate Best**: Statistician, power calculation, study design, data analysis. **Michael Nugent**: Developed project, secured funding, completion of IRAS form, secured ethical approval, writing of the protocol, writing patient information sheets, taking of 3D photos, volume change analysis, preparation of the final document.

## Data Availability

The data that support the findings of this study are available on request from the corresponding author. The data are not publicly available due to privacy or ethical restrictions.

## References

[cre2540-bib-0001] 3dMD, LLC . 2021. *3dMD… Welcome to the 4D Revolution*. http://www.3dMD.com

[cre2540-bib-0002] Agostinho, C. N. , da Silva, V. C. , Maia Filho, E. M. , Cruz, M. L. , & Bastos, E. G. (2014). The efficacy of 2 different doses of dexamethasone to control postoperative swelling, trismus, and pain after third molar extractions. General Dentistry, 62(6), e1–e5. 25369393

[cre2540-bib-0003] Aldridge, K. , Boyadjiev, S. A. , Capone, G. T. , DeLeon, V. B. , & Richtmaster, J. T. (2005). Precision and error of three‐dimensional phenotypic measures acquired from 3dMD photogrammetric images. American Journal of Medical Genetics, 138A, 247–253. 16158436 10.1002/ajmg.a.30959 PMC4443686

[cre2540-bib-0004] Amin, M. M. , & Laskin, D. M. (1983). Prophylactic use of indomethacin for prevention of postsurgical complications after removal of impacted third molars. Oral Surgery, Oral Medicine, Oral Pathology, 55, 448–451. 10.1016/0030-4220(83)90227-x 6575332

[cre2540-bib-0005] Asutay, F. , Ozcan‐Kucuk, A. , Alan, H. , & Koparal, M. (2018). Three‐dimensional evaluation of the effect of low‐level laser therapy on facial swelling after lower third molar surgery: A randomized, placebocontrolled study. Nigerian Journal of Clinical Practice, 21(9), 1107–1113. 30156193 10.4103/njcp.njcp_38_18

[cre2540-bib-0006] British Association of Head and Neck Oncologists (2009). BAHNO Standards 2009. http://www.bahno.org.uk/wp-content/uploads/2014/03/BAHNO-STANDARDS-DOC09.pdf 10.1111/jop.13161 33655561

[cre2540-bib-0007] ElHag, M. , Coghlan, K. , Christmas, P. , Harvey, W. , & Harris, M. (1985). The anti‐inflammatory effects of dexamethasone and therapeutic ultrasound in oral surgery. British Journal of Oral and Maxillofacial Surgery, 23, 17–23. 3156621 10.1016/0266-4356(85)90074-9

[cre2540-bib-0008] Esen, E. , Tasar, F. , & Akhan, O. (1999). Determination of the anti‐inflammatory effects of methylprednisolone on the sequelae of third molar surgery. Journal of Oral and Maxillofacial Surgery, 57, 1201–1206. 10513866 10.1016/s0278-2391(99)90486-x

[cre2540-bib-0009] Holland, C. S. (1979). The development of a method of assessing swelling following third molar surgery. British Journal of Oral Surgery, 17, 104–114. 10.1016/s0007-117x(79)80037-2 298834

[cre2540-bib-0010] Ibikunle, A. A. , Adeyemo, W. L. , & Ladeinde, A. L. (2016). Effect of submucosal or oral administration of prednisolone on postoperative sequelae following surgical extraction of impacted mandibular third molar: A randomized controlled study. Nigerian Medical Journal, 57(5), 272–279. 27833246 10.4103/0300-1652.190599 PMC5036298

[cre2540-bib-0011] Kau, C. H. , Cronin, A. , Durning, P. , Zhurov, A. I. , Sandham, A. , & Richmond, S. (2006). A new method for the 3D measurement of postoperative swelling following orthognathic surgery. Orthodontics & Craniofacial research, 9(1), 31–37. 16420272 10.1111/j.1601-6343.2006.00341.x

[cre2540-bib-0012] Markiewicz, M. R. , Brady, M. F. , Ding, E. L. , & Dodson, T. B. (2008). Corticosteroids reduce postoperative morbidity after third molar surgery: A systematic review and meta‐analysis. Journal of Oral and Maxillofacial Surgery, 66(9), 1881–1894. 18718396 10.1016/j.joms.2008.04.022

[cre2540-bib-0013] Matsuda, M. , Kondo, S. , Seto, M. , Kita, R. , Mori, H. , & Moriyama, S. (2016). Three‐dimensional quantitative evaluation of the effect of local administration of dexamethasone on facial swelling after impacted mandibular third molar extraction. Journal of Dentistry & Oral Disorders, 2(7), 1036.

[cre2540-bib-0014] Milles, M. , & Desjardins, P. J. (1993). Reduction of postoperative facial swelling by low‐dose methylprednisolone: An experimental study. Journal of Oral and Maxillofacial Surgery, 51, 987–991. 8355105 10.1016/s0278-2391(10)80041-2

[cre2540-bib-0015] Milles, M. , Desjardins, P. J. , & Pawel, H. E. (1985). The facial plethysmograph: A new instrument to measure facial swelling volumetrically. Journal of Oral and Maxillofacial Surgery, 43, 346–352. 3857298 10.1016/0278-2391(85)90255-1

[cre2540-bib-0016] Mocan, A. , Kişnişci, R. , & Üçok, C. (1996). Stereophotogrammetric and clinical evaluation of morbidity after removal of lower third molars by two different surgical techniques. Journal of Oral and Maxillofacial Surgery, 54(2), 171–175. 8604065 10.1016/s0278-2391(96)90441-3

[cre2540-bib-0017] Nord, F. , Ferjencik, R. , Seifert, B. , Lanzer, M. , Gander, T. , Matthews, F. , Rücker, M. , & Lübbers, H. T. (2015). The 3dMD photogrammetric photo system in cranio‐maxillofacial surgery: Validation of interexaminer variations and perceptions. Journal of Cranio‐Maxillofacial Surgery, 43(9), 1798–1803. 26421470 10.1016/j.jcms.2015.08.017

[cre2540-bib-0018] Pappalardo, S. , Puzzo, S. , Cappello, V. , Mastrangelo, F. , Adamo, G. , Caraffa, A. , & Tetè, S. (2007). The efficacy of four ways of administrating dexamethasone during surgical extraction of partially impacted lower third molars. European Journal of Inflammation, 5(3), 151–158.

[cre2540-bib-0019] Saravanan, K. , Kannan, R. , John, R. R. , & Kumar, C. N. (2016). A single pre operative dose of sub mucosal dexamethasone is effective in improving post operative quality of life in the surgical management of impacted third molars: A comparative randomised prospective study. Journal of Maxillofacial and Oral Surgery, 15(1), 67–71. 26929555 10.1007/s12663-015-0795-0 PMC4759017

[cre2540-bib-0024] Schache, A., Kerawala, C., Ahmed, O., Brennan, P. A., Cook, F., Garrett, M., Homer, J., Hughes, C., Mayland, C., Mihai, R., Newbold, K., O'Hara, J., Roe, J., Sibtain, A., Smith, M., Thavaraj, S., Weller, A., Winter, L., Young, V., & Winter, S. C. (2021). British Association of Head and Neck Oncologists (BAHNO) standards 2020. Journal of Oral Pathology & Medicine: Official Publication of the International Association of Oral Pathologists and the American Academy of Oral Pathology, 50(3), 262–273. 10.1111/jop.13161 33655561

[cre2540-bib-0020] Steele, A. C. , & Nugent, M. (2011). Retrospective audit of head and neck patients receiving dental extractions prior to radiotherapy. British Journal of Oral and Maxillofacial Surgery, 49(Suppl 1), S104.

[cre2540-bib-0021] Ullah, R. (2014). *The validity of 3dMD Vultus in predicting soft tissue morphology following orthognathic surgery* [Doctoral dissertation]. University of Birmingham.

[cre2540-bib-0022] Ullah, R. , Turner, P. J. , & Khambay, B. S. (2015). Accuracy of three‐dimensional soft tissue predictions in orthognathic surgery after Le Fort I advancement osteotomies. British Journal of Oral and Maxillofacial Surgery, 53(2), 153–157. 25432431 10.1016/j.bjoms.2014.11.001

